# Failure To Detect Prion Infectivity in Ticks following Prion-Infected Blood Meal

**DOI:** 10.1128/mSphere.00741-20

**Published:** 2020-09-02

**Authors:** Ronald A. Shikiya, Anthony E. Kincaid, Jason C. Bartz, Travis J. Bourret

**Affiliations:** a Department of Medical Microbiology and Immunology, Creighton University, Omaha, Nebraska, USA; b Pharmacy Science, Creighton University, Omaha, Nebraska, USA; Colorado State University

**Keywords:** chronic wasting disease, pathogenesis, prion

## Abstract

Chronic wasting disease (CWD) is an emerging and fatal contagious prion disease that affects cervids, including mule deer, white-tailed deer, black-tailed deer, red deer reindeer, elk, and moose. CWD prions are widely distributed throughout the bodies of CWD-infected animals and are found in the nervous system, lymphoid tissues, muscle, blood, urine, feces, and antler velvet. The mechanism of CWD transmission in natural settings is unknown. Potential mechanisms of transmission include horizontal, maternal, or environmental routes. Due to the presence of prions in the blood of CWD-infected animals, the potential exists for invertebrates that feed on mammalian blood to contribute to the transmission of CWD. The geographic range of the Rocky Mountain Wood tick, Dermancentor andersoni, overlaps with CWD throughout the northwest United States and southwest Canada, raising the possibility that *D. andersoni* parasitization of cervids may be involved in CWD transmission. We investigated this possibility by examining the blood meal of *D. andersoni* that fed upon prion-infected hamsters for the presence of prion infectivity by animal bioassay. None of the hamsters inoculated with a *D. andersoni* blood meal that had been ingested from prion-infected hamsters developed clinical signs of prion disease or had evidence for a subclinical prion infection. Overall, the data do not demonstrate a role for *D. andersoni* in the transmission of prion disease.

**IMPORTANCE** Chronic wasting disease (CWD) is an emerging prion disease that affects cervids, including mule deer, white-tailed deer, black-tailed deer, red deer reindeer, elk, and moose. The mechanism of CWD transmission in unknown. Due to the presence of prions in the blood of CWD-infected animals, it is possible for invertebrates that feed on cervid blood to contribute to the transmission of CWD. We examined the blood meal of *D.* andersoni, a tick with a similar geographic range as cervids, that fed upon prion-infected hamsters for the presence of prion infectivity by animal bioassay. None of the *D. andersoni* blood meals that had been ingested from prion-infected hamsters yielded evidence of prion infection. Overall, the data do not support a role of *D. andersoni* in the transmission of prion disease.

## Abstract (precis)

Chronic wasting disease (CWD) is an emerging prion disease that affects cervids, including mule deer, white-tailed deer, black-tailed deer, red deer reindeer, elk, and moose. The mechanism of CWD transmission in unknown. Due to the presence of prions in the blood of CWD-infected animals, it is possible for invertebrates that feed on cervid blood to contribute to the transmission of CWD. We examined the blood meal of *D.* andersoni, a tick with a similar geographic range as cervids, that fed upon prion-infected hamsters for the presence of prion infectivity by animal bioassay. None of the *D. andersoni* blood meals that had been ingested from prion-infected hamsters yielded evidence of prion infection. Overall, the data do not support a role of *D. andersoni* in the transmission of prion disease.

## INTRODUCTION

Chronic wasting disease (CWD) is a contagious prion disease that affects cervids, including mule deer, white-tailed deer, black-tailed deer, elk, reindeer, red deer, and moose (1–3). Like other prion diseases, CWD is an invariably fatal neurodegenerative disorder with an incubation period that ranges from years to decades. CWD was originally identified in captive deer in Colorado in 1967 and has spread geographically throughout North America; it has now been identified in captive and/or wild populations of cervids in 26 states and 3 Canadian provinces (4; https://www.usgs.gov/centers/nwhc/science/expanding-distribution-chronic-wasting-disease?qt-science_center_objects=0#qt-science_center_objects). More recently, CWD has been identified in free-ranging reindeer, red deer, and moose in Norway and moose in Sweden and Finland (5, 6). The mechanism of spread of CWD is not known but may occur by animal exposure to environmental sources of prions such as soil, blood, urine, feces, decaying carcasses, or maternal transmission (7–14).

The specific role of blood in prion pathogenesis is not known. Prions can be detected in blood during the course of disease and a blood transfusion from an infected animal can transmit a prion disease to a naive animal. Sheep naturally infected with scrapie, a prion disease that affects sheep and goats, or experimentally infected with BSE can transmit prion disease to naive sheep (15–17). Prions can be directly detected in mammalian blood using ultrasensitive methods of prion detection such as protein misfolding cyclic amplification (PMCA) and real-time quaking induced conversion (RT-QuIC). For instance, blood from deer infected with CWD contains RT-QuIC seeding activity and blood from prion-infected hamsters contains RT-QuIC and PMCA seeding activity (18–21). While infectivity or seeding activity can be detected in several host strain combinations, the contribution of prionemia to the transmission, pathogenesis, and ecology of prion disease is unknown (22).

**FIG 1:**
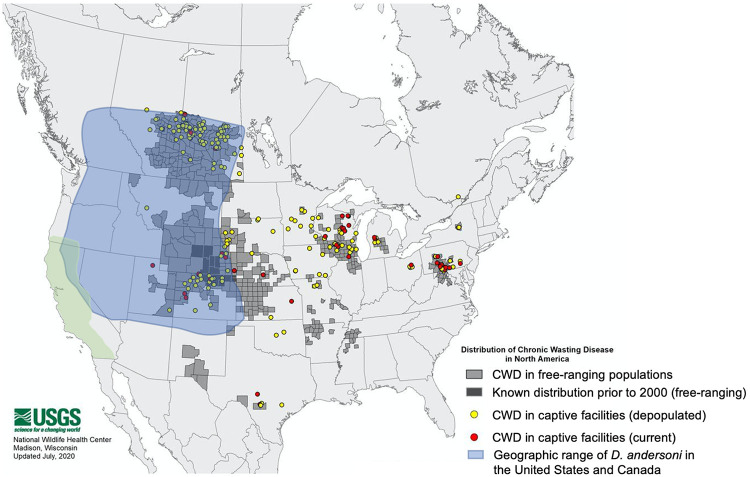
Geographic range of *D. andersoni* and distribution of CWD cases.

The Rocky Mountain Wood tick, Dermancentor andersoni, is a vector of several bacterial pathogens (Rickettsia rickettsii, Coxiella burnetii, Francisella tularensis, and Anaplasma marginale) and viral pathogens (Colorado tick fever virus and Powassan virus) and can cause tick paralysis (23–28). The geographic range of *D. andersoni* overlaps with CWD throughout the northwest United States and southwest Canada (Fig. 1). In this region, *D. andersoni* parasitize a variety of cervids during their adult stage and can concentrate blood from a bloodmeal up to 300%, raising the possibility that they may concentrate prion infectivity and serve as vectors of CWD (29–31). Here, we report the results of a set of experiments designed to test the hypothesis that *D. andersoni* serves as a vector for the spread of a prion disease.

## RESULTS

### Extranasal inoculation of hamsters with HY TME.

**FIG 2:**
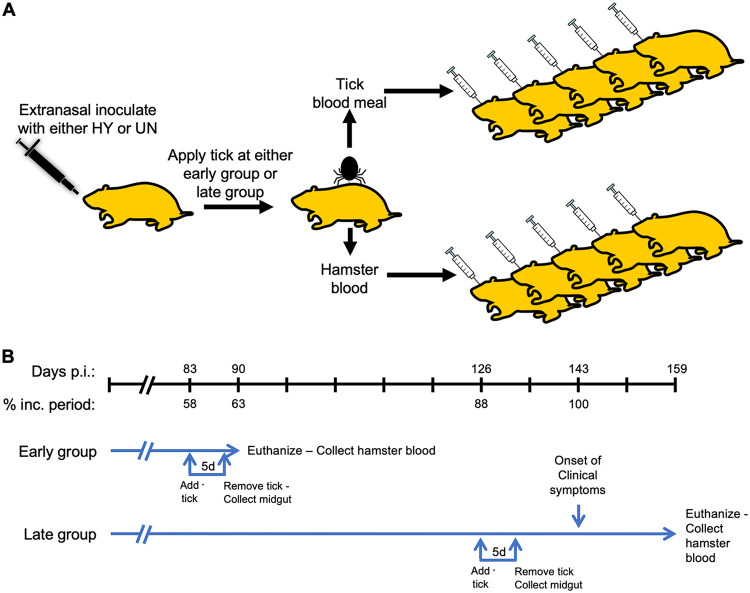
(A) Outline of experimental design. Groups of five hamsters were extranasally inoculated with either uninfected (UN) or hyper-infected (HY-infected) brain homogenate. (B) In the early group, ticks were allowed to feed on either the UN or HY-infected hamsters at 83 days p.i. for 5 days; they were then removed, and the midgut contents were collected. At 90 days p.i., these animals were euthanized, and blood was collected. Similarly, in the late group, ticks were allowed to feed on hamsters at 126 days p.i. for 5 days and then removed, and the midgut contents were collected. Blood from hamsters in the late group was collected at terminal disease. The tick midgut contents and blood from the early and late time points were intracerebrally inoculated into groups of five hamsters to determine whether they contained prion infectivity.

Ten hamsters were extranasally inoculated with uninfected brain homogenate, and 10 hamsters were extranasally inoculated with hyper-infected (HY-infected) brain homogenate. Five uninfected and five HY-infected animals had *D. andersoni* nymphs applied to them on day 83 postinfection (p.i.). The nymphs were removed on day 88 p.i., and the animals were euthanized on day 90 p.i.; these animals were designated as the “early group” (Fig. 2A). For the 10 remaining hamsters in the “late group” (5 infected, 5 uninfected), nymphs were applied at day 126 p.i. and removed at day 131 p.i. (Fig. 2A). All of the HY-infected animals in the late group developed clinical signs of hyperexcitability and ataxia at 143 ± 3 days postinfection, and none of the mock-infected hamsters developed clinical signs (Fig. 2A). All of the animals were euthanized at day 159 p.i. (Fig. 2A). The percent incubation periods reported in Fig. 2 are based on a 143-day incubation period. Overall, we collected midgut contents from engorged *D. andersoni* nymphs that fed on either uninfected or HY-infected animals about midway through, or just prior to, the onset of clinical signs to determine whether midgut contents from ticks contained prion infectivity.

### Animal bioassay of tick midgut contents and host hamster whole blood.

Tick midgut contents or whole blood from uninfected or HY-infected hamsters from either the early or late time group (Fig. 2B) were intracerebrally (i.c.) inoculated into five recipient hamsters to determine whether tick midgut contents or whole blood contained detectable prion infectivity (Fig. 2A). Importantly, the tick midgut samples were compared to the whole blood samples that were collected from the same animal. In other words, the tick midgut contents that were i.c. inoculated into five hamsters fed from the same animal that whole blood was collected from and i.c. inoculated into five separate recipient hamsters (Fig. 2A; Table 1). As a positive control, five hamsters were i.c. inoculated with a 1% (wt/vol) HY-infected brain homogenate, resulting in all of the animals developing clinical signs of hyperexcitability and ataxia at 61 ± 3 days p.i. (Table 1). None of the hamsters inoculated with tick midgut contents or blood from uninfected hamsters developed clinical signs of disease by 500 or 550 days p.i. (Table 1). Two of the five hamsters i.c. inoculated with blood collected from HY-infected late-group hamsters developed hyperexcitability and ataxia consistent with HY-infection and one intercurrent death at day 167 that was clinically negative (Fig. 2A; Table 1). All other hamsters i.c. inoculated with either tick midgut contents or blood from the early or late HY-infected group remained asymptomatic for 500 or 550 days p.i. (Table 1). Overall, under the conditions tested, tick midgut contents do not contain sufficient prion infectivity to cause clinical disease when inoculated into hamsters.

**TABLE 1:** Incubation period and attack rate of hamsters intracerebrally inoculated with blood or midgut homogenate from animals extranasally inoculated with either uninfected or HY-infected brain homogenate

Inoculum	A/I^*a*^	Incubation period^*b*^
HY-infected brain homogenate, 1% (wt/vol)	5/5	61 ± 3
		
Cardiac blood, early group		
UN#1	0/5	>500
HY#1	0/5	>500
HY#2	0/5	>500
HY#3	0/5	>500
		
Cardiac blood, late group		
UN#1	0/5	>500
HY#1	0/5	>500
HY#2	2/4^*c*^	238, 354
HY#3	0/5	>500
		
Tick midgut homogenate, early group		
UN#2	0/5	>550
HY#4	0/5	>550
HY#5	0/5	>550
HY#6	0/5	>550
		
Tick midgut homogenate, late group		
UN#2	0/5	>500
HY#4	0/5	>500
HY#5	0/5	>500
HY#6	0/5	>500

a A/I, affected/inoculated.

b Expressed in days p.i.

c There was one intercurrent death at 167 days p.i.

### Confirmation of clinical diagnosis of prion disease.

**FIG 3:**
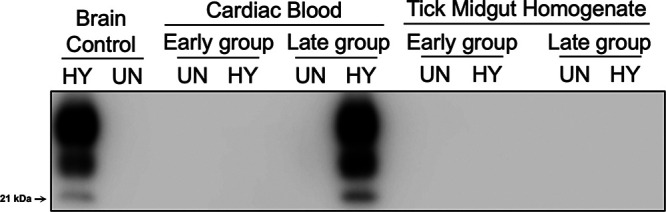
Confirmation of clinical diagnosis of animals inoculated with either tick midgut contents or hamster blood. Western blot analysis of PK-digested brain homogenate from either hyper-infected (HY-infected) or uninfected (UN) positive and negative controls, respectively, or from hamsters inoculated with either cardiac blood or tick midgut contents from UN or HY-infected hamsters at either early or late times. The molecular weight marker is indicated on left of panel. This experiment was repeated a minimum of three times with similar results.

**FIG 4:**
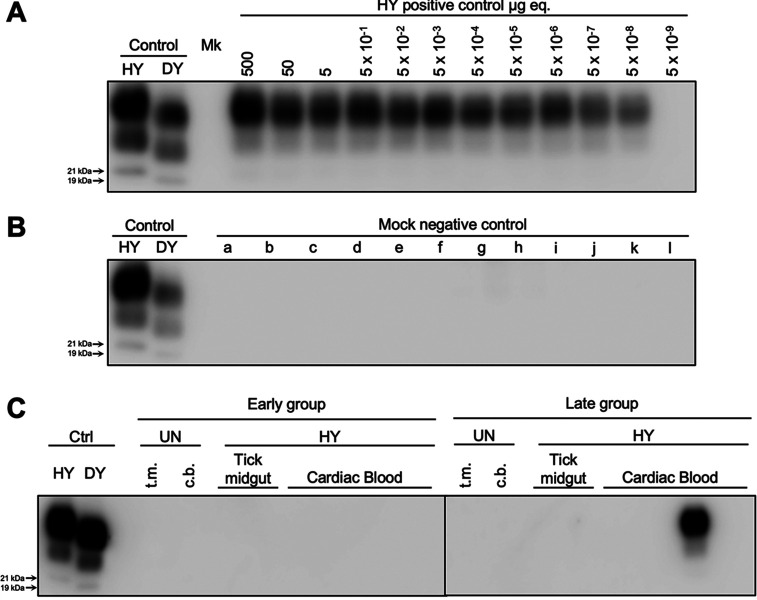
PMCA analysis of brain material of hamsters inoculated with either tick midgut contents or blood failed to detect PrP^Sc^. (A to C) Western blot analysis of PMCA reactions seeded with 10-fold serial dilutions of HY-infected positive-control brain (A), mock-infected negative-control brain (B), or brain material from hamsters infected with either tick midgut (t.m.) contents or blood collected by cardiac puncture (c.b.) from uninfected (UN) or HY-infected animals from the early and late group (C). This experiment was repeated a minimum of three times with similar results.

Western blot and 96-well immunoassay analyses were used to confirm the clinical diagnosis of disease by determining the presence or absence of PrP^Sc^ in brain material from the hamsters listed in Table 1. Brain homogenate from hamsters that did not develop clinical signs of prion infection did not contain detectable PrP^Sc^ as determined by either Western blotting (Fig. 3) or 96-well immunoassay (see Fig. S1 in the supplemental material). Brain homogenate from animals that developed clinical signs of hyperexcitability and ataxia (Table 1) contained PrP^Sc^ with an unglycosylated polypeptide that migrated at 21 kDa, a finding consistent with infection with HY TME (Fig. 3). To determine whether a subclinical HY TME infection had occurred, we used PMCA to determine whether PrP^Sc^ was present in brain from animals that were below the limit of detection of Western blotting or 96-well immunoassay. Positive-control PMCA reactions were seeded with 10-fold serial dilutions of HY-infected brain and, after one round of PMCA, PrP^Sc^ amplification was detected in every HY TME dilution down to 10^−9^ (Fig. 4A). Negative-control PMCA reactions seeded with uninfected brain homogenate did not result in detection of PrP^Sc^ (Fig. 4B). PMCA reactions seeded with brain homogenate from hamsters i.c. inoculated with tick midgut homogenate from ticks that fed on either uninfected or HY-infected animals or blood from these same animals at either the early or late group (Table 1) that did not develop clinical disease did not result in amplification of detectable PrP^Sc^ (Fig. 4C; Table S1) in contrast to animals that did develop clinical disease resulted in amplification of PrP^Sc^ (Fig. 4A; Table S1). Overall, using both conventional and ultrasensitive methods of PrP^Sc^ detection, we did not find evidence of a subclinical prion infection from animals inoculated with midgut contents from ticks that fed on HY-infected hamsters.

10.1128/mSphere.00741-20.1 FIG S1 Confirmation of clinical diagnosis of animals inoculated with either tick midgut contents listed in Table 1. Proteinase K-digested brain homogenates from hamsters inoculated with either tick midgut contents or blood from animals that were mock (UN) inoculated or HY-infected at the early or late time point postinfection, as indicated in Fig. 2. Panels in this figure were repositioned for clarity and originated from the same exposure of the same 96-well plate. This experiment was repeated a minimum of three times with similar results. Download FIG S1, TIF file, 2.8 MB. Copyright © 2020 Shikiya et al. 2020 Shikiya et al. This content is distributed under the terms of the Creative Commons Attribution 4.0 International license.

10.1128/mSphere.00741-20.2 TABLE S1 Summary of PMCA of brain homogenates from animals inoculated with cardiac blood or tick midgut homogenate. Download Table S1, DOCX file, 0.02 MB. Copyright © 2020 Shikiya et al. 2020 Shikiya et al. This content is distributed under the terms of the Creative Commons Attribution 4.0 International license.

## DISCUSSION

This study was carried out to determine whether *D. andersoni* can acquire prions from an infected mammalian host and potentially serve as a vector for prion transmission. The rationale for this study was based on the extensive overlap of the ecological range of *D. andersoni* with cases of CWD among cervids in the northwest United States and southwest Canada (Fig. 1). The ability of *D. andersoni* to serve as a competent vector for prions could contribute to the expansion of the range of CWD. The ecological range of *D. andersoni* and many other hard-bodied ticks is known to be expanding and, along with this expansion, there is an increased incidence of various tick-borne diseases. The reason for the tick range expansion is not known, but it may be due to changes in climate that could result in an increased success of oviposition by engorged females, which has previously been shown under controlled laboratory conditions (32).

*D. andersoni* have a three-stage life cycle that requires blood meals from a variety of small mammals during larval and nymph stages and from large mammals such as deer and elk during the adult stage. Syrian hamsters have been used as a model system to assess the vector competence of D. andersoni and Dermacentor variabilis, a closely related tick species, for a variety of bacterial and viral pathogens (33–35). In this study, we used a well-established model of prion infection to determine the ability of *D. andersoni* nymphs to acquire prions from infected animals. For the experiments described here, *D. andersoni* nymphs were fed to repletion on both uninfected and prion-infected Syrian hamsters. Blood collected from prion-infected hamsters in the late group was capable of infecting naive hamsters (Table 1). During feeding, *D. andersoni* concentrate blood meals ingested from mammalian hosts by secreting up to 80% of water through the salivary gland and anus (30). Therefore, the concentration of prions in midgut homogenates collected from engorged ticks could be increased up to 300% compared to prion concentrations in undiluted whole blood of infected hamsters used in the animal bioassay experiments (29). However, midgut homogenates derived from *D. andersoni* nymphs that had fed to repletion on prion-infected hamsters did not possess infectious prions as determined by animal bioassay.

The lack of detectable prion infectivity in the midgut contents of blood-fed *D. andersoni* nymphs may be due to the fact that arthropods, including hard-bodied ticks, do not have a PrP^C^ homologue encoded in their genomes (36, 37), which may prevent the amplification of PrP^Sc^ within the midgut lumen or midgut epithelial cells of blood-fed nymphs (38). The lumen of the tick midgut is slightly acidic (pH 6.5 to 6.8), harbors hemolysins that lyse erythrocytes, and is generally considered to be a favorable environment for pathogenic microorganisms. Lysed erythrocytes and other components of the blood meal are endocytosed and sequestered in highly acidic endo/lysosomal vesicles within midgut epithelial cells, where they are degraded by cysteine and aspartic acid peptidases (39). The lack of detectable prion infectivity in the midguts of *D. andersoni* nymphs could be the result of their degradation by tick peptidases or through altered conformation within the highly acidic endo/lysosomal vesicles in midgut epithelial cells. An additional possibility is that other components of the blood meal, such as toxic by-products arising from the hemolysis of erythrocytes inactivate PrP^Sc^. In addition, the tick midgut is a highly oxidative environment due to the enzymatic production of reactive oxygen species (ROS) by dual oxidase enzymes (40, 41). A previous study provided evidence for ROS inactivation of infectious 263K prions produced by neutrophils and by a weakly acidic aqueous solution of hypochlorous acid, presumably due to oxidative modifications to sulfhydryl groups of cysteines (42). Additional investigations will be required to determine the impact of these environmental factors within the tick midgut on prions.

The failure to detect prion infectivity in tick midgut contents could be a consequence of the limitations of animal bioassay. We chose to assay the contents of the tick midgut for prions by i.c. inoculation as this is the most sensitive method of inoculation to determine whether *bona fide* prion infectivity was present (43). One interpretation of this data is that tick midgut contents do not contain prion infectivity; however, one cannot exclude the possibility that prions are present in tick midgut contents but were not detected due to the limitations of animal bioassay. Detection of prion infectivity in whole blood has been inconsistently observed in numerous host/strain combinations (16, 17, 44–46). Prion infectivity in blood is mainly associated with buffy coat cells, and bioassay of whole blood may be a less reliable means of transmitting prion infectivity since the buffy coat cells are dispersed in the blood lowering the effective titer per volume (47). Although it is known that hamsters infected with HY TME have prionemia, it may be that HY TME does not fully recapitulate the pathogenesis of CWD. (48). In addition, we did not examine tick midgut contents for prion infectivity that fed on animals during the clinical phase of disease and therefore cannot exclude the possibility that ticks may harbor prion infectivity that feed on clinically affected animals. It is also possible that sampling a small volume of blood per animal (<0.5%), and a limited number of engorged nymphs collected from each animal (<10) in combination with a limited number of animals per group (*n* = 5) may result in a false-negative result.

Differences between what is known about operational versus functional prion infectivity complicate interpretation of negative results. Prion infectivity is operationally defined by a response in animals under defined conditions; however, it has long been recognized that a bioassay may not measure all of the infectious (e.g., functional) particles present in an inoculum (49). Seeded amplification assays such as PMCA and RT-QuIC can reliably and specifically detect prion seeding activity that is several orders of magnitude more sensitive than an animal bioassay (50–55). The relationship between prion infectivity in animals and *in vitro* seeding activity is only beginning to be understood (56). Detection of prions in blood with PMCA and RT-QuIC from hosts in the preclinical phase of disease before the reliable detection of infectivity in experimental and naturally occurring prion disease is well documented (19–21, 57–62) and raises the possibility that this *in vitro* seeding activity represents *bona fide* infectious prion particles. Since factors that affect the efficiency of prion infection are not fully understood (63, 64), future studies using these ultrasensitive methods of prion detection from bloodmeals may provide valuable insight into the role of arthropods in the ecology of prion disease.

## MATERIALS AND METHODS

### Ethics statement.

All procedures involving hamsters were approved by the Creighton University Institutional Animal Care and Use Committee and comply with the *Guide for the Care and Use of Laboratory Animals*.

### Animal bioassay.

Male Syrian hamsters (10 to 11 weeks old) were extranasally inoculated with 100 μl of 10% (wt/vol) brain homogenate from either uninfected or HY TME-infected hamsters as previously described (65). Male 3- to 4-week-old Syrian hamsters were intracerebrally inoculated with 25 μl of either homogenized tick midgut contents (the preparation is described below), undiluted whole blood, or brain homogenate, as previously described (66). Animals were monitored 3 days per week for the onset of neurological disease. The incubation period was calculated as the number of days between inoculation and the onset of clinical signs of prion disease.

### Tick feeding.

Pathogen-free *D. andersoni* nymphs were acquired from the Oklahoma State Tick Rearing facility and stored in a desiccator at ∼22°C with 80% relative humidity prior to feeding on Syrian hamsters. Feeding capsules were made from 20-ml syringes and adhered to the shaved dorsal surface of hamsters using cyanoacrylate glue. Fifteen *D. andersoni* nymphs were added to each feeding capsule and allowed to feed on hamsters for up to 5 days, which had been empirically determined to be maximum length of time for adherence of the feeding capsule to hamsters. Unfed ticks were discarded, and midguts dissected from engorged ticks (6 to 14 ticks per hamster) collected from the same animal were pooled in a 1.5-ml microcentrifuge tube, and homogenized with a pestle and vigorous pipetting to a final volume of 125 μl of phosphate-buffered saline (PBS; pH 7.4).

### Protein misfolding cyclic amplification.

PMCA was performed as previously described, with modifications (67). Briefly, samples (*n* ≥ 3) in PMCA conversion buffer (supplemented with heparin, NaCl, and digitonin) were placed in a Qsonica Q700MPX sonicator (Newtown, CT) with an average output of 230 W during each sonication cycle. A single round of PMCA consisted of 432 cycles of a 1-s sonication, followed by an incubation of 9 min and 59 s at 37°C. The ratio of seed to uninfected brain homogenate was 1:20. Aliquots of (*n* ≥ 3) of HY TME were included as a positive control. Aliquots (*n* ≥ 8) of uninfected brain homogenate (Mock) were included in all rounds of PMCA as a negative control.

### 96-well immunoassay.

The 96-well immunoassay was performed as described previously (68). Briefly, each well of the MultiScreen filter plate (Millipore-Sigma, Burlington, MA) was activated with 200 μl of methanol and removed by pipette aspiration. The wells were washed with Tween/Tris-buffered saline (TTBS) and centrifuged at 1,500 × *g* in a Centra GP8R tabletop centrifuge (Thermo IEC, Waltham, MA). Brain homogenates were incubated with proteinase K (PK) at a final concentration of 50 μg/ml (Roche Diagnostics Corporation, Indianapolis, IN), diluted into 200 μl of DPBS, added to the well, and centrifuged at 1,500 × *g*. To block endogenous peroxidase activity, 200 μl of 0.3% hydrogen peroxide (Millipore-Sigma) in methanol was added to each well. To denature the prion protein, 200 μl of 3 M guanidine thiocyanate (Millipore-Sigma) was added to each well. Each well was incubated with 5% (wt/vol) nonfat dry milk in TTBS (Bio-Rad Laboratories, Hercules, CA). Mouse monoclonal anti-PrP antibody 3F4 (0.1 μg/ml; Millipore-Sigma) was used to detect hamster prion protein. The plate was developed with SuperSignal West Femto maximum-sensitivity substrate (Pierce, Rockford, IL), and imaged using a Li-Cor Odyssey Fc imaging system (Li-Cor, Lincoln, NE), and PrP abundance was quantified by using Li-Cor Image Studio Software v.1.0.36.

### Western blot analysis.

Detection of PrP^Sc^ by Western blotting was performed as previously described (69). Briefly, PMCA reaction samples were incubated with PK at a final concentration of 50 μg/ml (Roche Diagnostics Corporation, Indianapolis, IN) at 37°C for 60 min with agitation. Digestion was terminated by boiling samples at 100°C for 10 min in sample loading buffer (4% [wt/vol] SDS, 2% [vol/vol] β-mercaptoethanol, 40% [vol/vol] glycerol, 0.004% [wt/vol] bromophenol blue, and 0.5 M Tris buffer [pH 6.8]) immediately prior to size fractionation on 4 to 12% bis-Tris-acrylamide gels (NuPAGE; Invitrogen, Carlsbad, CA), followed by transfer to a polyvinylidene difluoride membrane (Immobilon P; Millipore-Sigma). Membranes were incubated with 5% (wt/vol) nonfat dry milk in TTBS (Bio-Rad) for 30 min. Mouse monoclonal anti-PrP antibody 3F4 (0.1 μg/ml; Millipore-Sigma) was used to detect hamster prion protein. Western blots were developed with SuperSignal West Femto maximum-sensitivity substrate and imaged using the Li-Cor Odyssey Fc imaging system, and PrP abundance was quantified using Li-Cor Image Studio Software v.1.0.36.
