# Supplementary figures and images for: Failure To Detect Prion Infectivity in Ticks following Prion-Infected Blood Meal
Source: mSphere. 2020 Sep 2;5(5):e00741-20. doi: 10.1128/mSphere.00741-20 (PMC7471009; doi:10.1128/mSphere.00741-20)

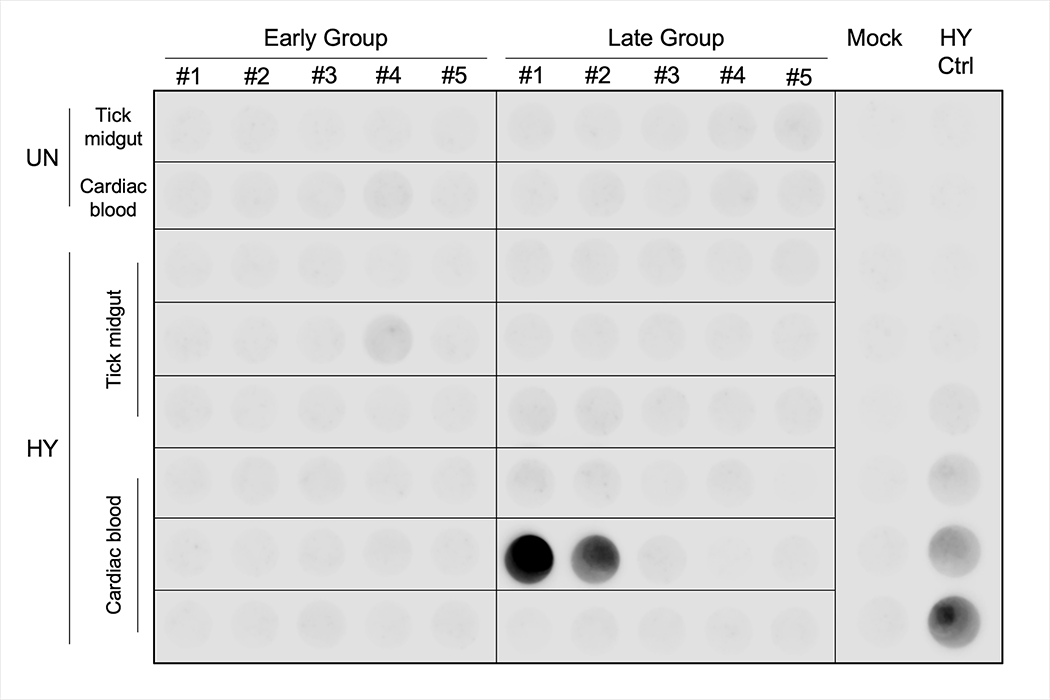

Supplement: FIG S1 [file mSphere.00741-20-sf001.tif]
