# Supplementary material for: Failure To Detect Prion Infectivity in Ticks following Prion-Infected Blood Meal
Source: mSphere. 2020 Sep 2;5(5):e00741-20. doi: 10.1128/mSphere.00741-20 (PMC7471009; doi:10.1128/mSphere.00741-20)
Supplement: TABLE S1 [file mSphere.00741-20-st001.docx]

**Supplemental table 1**. Summary of PMCA of brain homogenates from animals inoculated with cardiac blood or tick midgut homogenate.

^a^ number of animals positive for PMCA seeding activity / number of animals tested per group

| Brain material used as PMCA seed from animals inoculated with: | PMCA results |
| --- | --- |
| Cardiac blood 90 d. p.i. UN #1 | 0/5^a^ |
| Cardiac blood 90 d. p.i. HY #1 | 0/5 |
| Cardiac blood 90 d. p.i. HY #2 | 0/5 |
| Cardiac blood 90 d. p.i. HY #3 | 0/5 |
| Cardiac blood 159 d. p.i. UN #2 | 0/5 |
| Cardiac blood 159 d. p.i. HY #4 | 0/5 |
| Cardiac blood 159 d. p.i. HY #5 | 2/5 |
| Cardiac blood 159 d. p.i. HY #6 | 0/5 |
| Tick midgut homog. 88 d. p.i. UN #1 | 0/5 |
| Tick midgut homog. 88 d. p.i. HY #1 | 0/5 |
| Tick midgut homog. 88 d. p.i. HY #2 | 0/5 |
| Tick midgut homog. 88 d. p.i. HY #3 | 0/5 |
| Tick midgut homog. 131 d. p.i. UN #2 | 0/5 |
| Tick midgut homog. 131 d. p.i. HY #4 | 0/5 |
| Tick midgut homog. 131 d. p.i. HY #5 | 0/5 |
| Tick midgut homog. 131 d. p.i. HY #6 | 0/5 |
